# Association Between Collagen-Based Hemostatic Patch Use and Patient-Reported Recovery After Bilateral Thyroidectomy: A Retrospective Cohort Study

**DOI:** 10.3390/jcm15186936

**Published:** 2026-09-08

**Authors:** Horng-Shiuan See, Kian-Hwee Chong, Yu-Tien Chen, Jia-Hui Chen, Chieh-Wen Lai

**Affiliations:** 1 Department of Surgery, Taipei Tzu Chi Hospital, Buddhist Tzu Chi Medical Foundation, New Taipei City 23142, Taiwan; 2 School of Medicine, Tzu Chi University, Hualien 97004, Taiwan

**Keywords:** thyroidectomy, hemostatic patch, patient-reported outcomes, postoperative recovery, retrospective cohort

## Abstract

**Background/Objectives:** Topical hemostatic agents are widely used in thyroid surgery, but their clinical value remains uncertain. We evaluated whether intraoperative use of a collagen-based hemostatic patch during bilateral thyroidectomy was associated with patient-reported recovery and postoperative complications. **Methods:** This retrospective cohort study included 193 patients undergoing bilateral thyroidectomy (98 received Hemopatch and 95 underwent conventional hemostasis without a patch). Pain and swallowing scores were summarized as medians with interquartile ranges and compared using two-sided Mann–Whitney U tests. Unadjusted mean differences with 95% percentile-bootstrap confidence intervals based on 10,000 independent-group resamples were reported as effect estimates. Multivariable proportional-odds ordinal logistic regression adjusting for age, sex, operative time, intraoperative blood loss, and pathology was performed as a sensitivity analysis. **Results:** Major postoperative complications were infrequent, with no statistically significant between-group differences. Patch use was associated with lower pain scores on POD7 (mean difference −0.34, 95% CI −0.59 to −0.09; *p* = 0.0008), POD30 (−0.11, 95% CI −0.22 to 0.01; *p* = 0.0223), and POD90 (−0.12, 95% CI −0.18 to −0.05; *p* = 0.0005), but not POD1. Swallowing disturbance was also associated with lower scores on POD7 (−0.35, 95% CI −0.67 to −0.03; *p* = 0.0068), POD30 (−0.17, 95% CI −0.38 to 0.05; *p* = 0.0272), and POD90 (−0.23, 95% CI −0.38 to −0.09; *p* = 0.0005), but not POD1. Scar satisfaction was associated with a higher mean score at 6 months (+0.46, 95% CI +0.21 to +0.71; *p* = 0.0018). Several associations remained significant after multivariable adjustment and Holm correction, although not all comparisons were robust to multiplicity adjustment. **Conclusions:** Collagen-based hemostatic patch use was associated with modest differences in selected patient-reported recovery measures. The magnitude and clinical importance of these differences remain uncertain. No statistically significant differences in major complications were observed, but the study was not powered to establish comparative safety for uncommon events. These exploratory findings require confirmation in prospective multicenter studies.

## 1. Introduction

Thyroidectomy involves the surgical removal of all or part of the thyroid gland. The number of thyroidectomies performed worldwide is increasing because of the rising incidence of thyroid nodules and carcinoma [1,2]. Thyroidectomy is generally considered a safe procedure when performed by an experienced surgeon. However, it remains associated with clinically important complications, including postoperative hemorrhage, recurrent laryngeal nerve (RLN) injury, hypoparathyroidism, seroma formation, and wound infection [3,4,5]. Postoperative hemorrhage, which may lead to airway compromise, is a particularly critical complication and may necessitate reoperation [6,7].

Substantial improvements in surgical techniques have been made over the past two decades. Refinements in capsular dissection and in identification and preservation of the RLN and parathyroid glands have improved procedural safety. In addition, energy-based devices such as LigaSure and the harmonic scalpel have been developed and shown to reduce intraoperative blood loss and operative time while maintaining safety [8,9,10]. Despite these advances, achieving reliable hemostasis and minimizing postoperative morbidity remain important challenges in endocrine surgery [11,12].

Topical hemostatic agents, such as gelatin–thrombin matrices, oxidized cellulose, and collagen-based pads, have emerged as promising adjuncts that may improve intraoperative hemostasis and optimize the local surgical field [13,14]. Several prospective studies and meta-analyses have suggested that these agents may reduce minor bleeding and facilitate postoperative recovery, although their overall clinical value remains debated [15,16,17,18,19]. Their use in thyroid surgery has therefore attracted growing interest, particularly in the context of improving postoperative outcomes beyond conventional safety endpoints [20].

Accordingly, the potential value of adjunctive hemostatic agents may extend beyond technical hemostasis alone. In addition to conventional surgical outcomes, their use may be associated with patient-reported recovery measures such as pain, swallowing comfort, and scar satisfaction. We therefore conducted a retrospective cohort study to examine whether use of a collagen-based hemostatic patch during bilateral thyroidectomy was associated with differences in these recovery outcomes and major postoperative complications.

## 2. Materials and Methods

### 2.1. Study Design and Patient Population

This retrospective cohort study was conducted at Taipei Tzu Chi Hospital. The institutional surgical database initially identified 296 potentially eligible adults who underwent bilateral thyroidectomy between January 2019 and May 2021. During revision, we returned to the archived individual-level analytic data to verify the study cohort and reproduce the statistical analyses. Complete records sufficient for the revised analysis were retrievable for 193 patients, comprising 98 patients treated with a collagen-based hemostatic patch and 95 patients managed without a patch. Three Hemopatch records lacked postoperative hypoparathyroidism data. The complete archived patient-level dataset underlying the previously performed propensity-score matching analysis could not be fully reconstructed with sufficient certainty; therefore, no new propensity-score matching was performed and the revised analysis used these 193 verifiable records as an unmatched retrospective cohort. The study was conducted in accordance with the Declaration of Helsinki (as revised in 2013), and reporting followed the STROBE guidelines for observational studies. Patients were eligible if they were aged ≥18 years and underwent elective total or near-total thyroidectomy for benign or malignant disease. Patients with a history of neck surgery or concomitant parathyroidectomy were excluded. All procedures were performed by a single experienced endocrine surgeon (C.-W.L.) using standardized capsular dissection, systematic identification of the recurrent laryngeal nerve (RLN) and parathyroid glands, and vessel ligation.

### 2.2. Group Allocation and Perioperative Management

Patients were classified according to the intraoperative hemostatic strategy used in routine clinical practice (nonrandomized), reflecting surgeon preference and real-time assessment of the operative field. The patch group (*n* = 98) received a collagen-based hemostatic patch (Hemopatch; Baxter Healthcare, Deerfield, IL, USA) applied to the thyroid bed at the end of surgery, whereas the no-patch group (*n* = 95) underwent conventional hemostasis without patch use. No drain was placed in either group. Standardized anesthesia and perioperative care pathways were used. Patients were followed in the outpatient clinic for at least 6 months.

### 2.3. Data Collection and Outcome Measures

Demographic data (age and sex), operative parameters (operative time and intraoperative blood loss), pathology (benign or malignant disease), length of hospital stay, and postoperative outcomes were collected for all patients. The covariates used in the adjusted sensitivity models were age, sex, operative time, intraoperative blood loss, and malignant pathology. The primary outcomes were postoperative hemorrhage or hematoma requiring intervention, RLN injury (confirmed clinically and, if symptomatic, by laryngoscopy), and hypoparathyroidism. Transient hypoparathyroidism was defined as hypocalcemia requiring calcium and vitamin D supplementation that resolved within 3 months postoperatively, and permanent hypoparathyroidism as persistent hypocalcemia requiring ongoing supplementation beyond 3 months. Three Hemopatch records lacked hypoparathyroidism data; no imputation was performed, and this outcome was analyzed using available cases.

Secondary outcomes were postoperative pain and swallowing disturbance, each measured using a 10-point visual analog scale (VAS) at routine postoperative assessments on postoperative days 1, 7, 30, and 90, and scar satisfaction, measured using a 10-point VAS at 6 months postoperatively. These patient-reported outcomes were collected as part of standard postoperative assessment and documented in the medical record at each visit. No follow-up values for pain, swallowing, or scar satisfaction were missing. Higher scores indicated greater pain intensity, greater swallowing discomfort, and greater scar satisfaction, respectively.

### 2.4. Statistical Analysis

The originally submitted analysis used propensity-score matching. During revision, however, the complete archived individual-level dataset required to reproduce the original matching procedure was not fully retrievable. Because the original pair assignments and matching data could not be independently reconstructed, it was methodologically inappropriate to retain or recreate a matched analysis. No propensity-score matching was performed in the revised analysis; consequently, a matching algorithm, caliper, matching ratio, and post-matching balance threshold were not applicable. Descriptive absolute standardized mean differences (SMDs) for the available preoperative and operative characteristics are reported in Table 1 to show between-group imbalance, but they are not post-matching balance diagnostics. Distributional assumptions for continuous variables were assessed using the Shapiro–Wilk test together with inspection of the observed distributions. Approximately normally distributed continuous variables are presented as mean ± standard deviation (SD) and were compared using Welch’s t-test. Skewed continuous or ordinal variables are presented as median and interquartile range (IQR) and were compared using the two-sided Mann–Whitney U test. Categorical variables are presented as *n* (%) and were compared using Fisher’s exact test.

Pain and swallowing VAS scores demonstrated marked non-normality and floor effects, particularly at later postoperative time points; these outcomes were therefore analyzed using separate time-specific, unpaired rank-based comparisons rather than a joint repeated-measures or mixed-effects longitudinal model. Within-patient correlation across repeated time points was not modeled. Unadjusted mean differences (Hemopatch minus no patch) and 95% confidence intervals were estimated by percentile bootstrap resampling independently within each group (10,000 resamples) and are reported as effect estimates. Because comparisons were performed at four predefined time points for each symptom domain, nominal *p* values are reported as the primary exploratory results, with Holm-adjusted *p* values calculated separately within the pain and swallowing families as a sensitivity analysis for multiple testing. No pain, swallowing, or scar-satisfaction follow-up values were missing; each time-specific analysis used all available observations. Because the VAS distributions contained ties, Mann–Whitney *p* values used the asymptotic method.

Because treatment allocation was nonrandomized, multivariable proportional-odds ordinal logistic regression was performed as an adjusted sensitivity analysis for patient-reported outcomes. Separate models were fitted for each outcome and time point; these were not joint longitudinal models and did not estimate within-patient correlation across time points. Hemostatic patch use was entered as the exposure, with adjustment for age, sex, operative time, intraoperative blood loss, and histopathological diagnosis (benign vs. malignant). Adjusted common odds ratios (aORs) with 95% confidence intervals (CIs) were reported. For pain and swallowing scores, an aOR < 1 indicates lower odds of being in a higher symptom category; for scar satisfaction, an aOR > 1 indicates higher odds of being in a higher satisfaction category. The adjusted model for pain on postoperative day 90 was not considered estimable because all patients in the patch group reported a score of 0, resulting in complete separation. Adjusted models were not fitted for major complications because event counts were too small for reliable multivariable estimation. Three Hemopatch records lacked hypoparathyroidism data; no imputation was performed, and this safety outcome was analyzed using available cases.

No a priori sample-size or power calculation was performed. The analyses of patient-reported outcomes were exploratory, and the study may have been underpowered to detect clinically important differences in uncommon complications. All tests were two-sided, *p* values are displayed to four decimal places, and *p* < 0.05 was considered statistically significant for nominal analyses. Analyses were conducted using SPSS version 18.0 (IBM Corp., Armonk, NY, USA). The proportional-odds ordinal regression models and Holm-adjusted sensitivity analyses were reproduced in Python version 3.13.5 using SciPy version 1.17.0 and statsmodels version 0.14.6.

## 3. Results

### 3.1. Patient Demographics

Of the 296 potentially eligible patients initially identified in the institutional database, complete archived individual-level records sufficient for the revised analysis were retrievable for 193 patients: 98 in the Hemopatch group and 95 in the no-patch group (Table 1). Patients receiving Hemopatch were somewhat older (58.26 ± 12.13 vs. 55.19 ± 13.00 years; *p* = 0.0921; absolute SMD = 0.244) and included a lower proportion of men (19.4% vs. 26.3%; *p* = 0.3039; absolute SMD = 0.166). Operative time, intraoperative blood loss, and pathological diagnosis were similar between groups. Median hospital stay was 3 days in both groups (*p* = 0.0470; absolute SMD = 0.285); hospital stay is a postoperative characteristic and was not used as an adjustment covariate. These observed differences, particularly in age and sex, motivated the adjusted sensitivity analysis specified in Section 2.4.

### 3.2. Surgical Complications

Major postoperative complications were uncommon, and no statistically significant between-group differences were observed (Table 2). Postoperative hematoma occurred in 3 of 98 patients (3.1%) in the patch group and 4 of 95 (4.2%) in the no-patch group (risk difference −0.0115, 95% CI −0.0638 to 0.0405; Fisher’s exact *p* = 0.7180). RLN injury occurred in 4 of 98 (4.1%) and 5 of 95 (5.3%), respectively (risk difference −0.0118, 95% CI −0.0740 to 0.0497; *p* = 0.7448). Among evaluable patients, hypoparathyroidism occurred in 14 of 95 (14.7%) and 15 of 95 (15.8%), respectively (risk difference −0.0105, 95% CI −0.1158 to 0.0947; *p* = 1.0000). No cases of permanent hypoparathyroidism were identified, and no postoperative hematoma required reoperation. Because these events were infrequent and no a priori power calculation was performed, the absence of statistical significance should not be interpreted as evidence of safety equivalence.

### 3.3. Postoperative Pain

**Figure 1:**
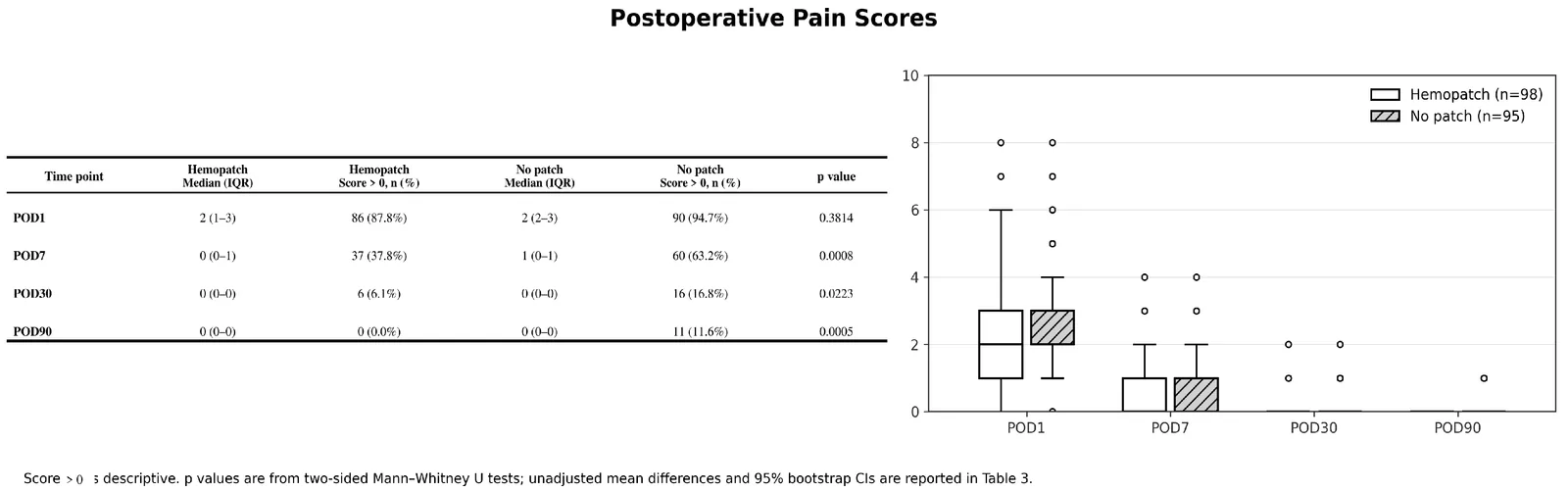
Postoperative pain scores during follow-up in the full unmatched cohort. The left summary reports median (IQR), the number (%) of patients with a score > 0, and the nominal between-group *p* value at each time point. Score > 0 is shown descriptively to make floor effects transparent; *p* values are from two-sided Mann–Whitney U tests using the full ordinal score distributions. Unadjusted mean differences with 95% bootstrap CIs are reported in Table 3. In the box plots, boxes represent the IQR, center lines represent medians, whiskers extend to the most extreme observations within 1.5 times the IQR, and points beyond the whiskers represent outliers. Hemopatch is shown as a white box and no patch as a hatched box. IQR, interquartile range.

Postoperative pain scores were markedly right-skewed with substantial floor effects at later follow-up (Figure 1). On postoperative day 1, the unadjusted mean difference was −0.19 (95% CI −0.66 to 0.27; Mann–Whitney *p* = 0.3814). Patch use was associated with lower pain score distributions on day 7 (mean difference −0.34, 95% CI −0.59 to −0.09; *p* = 0.0008), day 30 (−0.11, 95% CI −0.22 to 0.01; *p* = 0.0223), and day 90 (−0.12, 95% CI −0.18 to −0.05; *p* = 0.0005). After Holm adjustment across the four pain time points, the adjusted *p* values were 0.3814, 0.0023, 0.0446, and 0.0022, respectively. Because the floor effect produced identical medians at the later time points, Figure 1 also shows the proportion of patients with any residual pain (score > 0) descriptively. A score > 0 was present in 37 of 98 patients (37.8%) versus 60 of 95 (63.2%) on day 7, 6 of 98 (6.1%) versus 16 of 95 (16.8%) on day 30, and 0 of 98 (0%) versus 11 of 95 (11.6%) on day 90 in the patch and no-patch groups, respectively. These frequencies are descriptive; the inferential *p* values are based on the full ordinal score distributions, and effect estimates are summarized in Table 3.

### 3.4. Swallowing Disturbance

**Figure 2:**
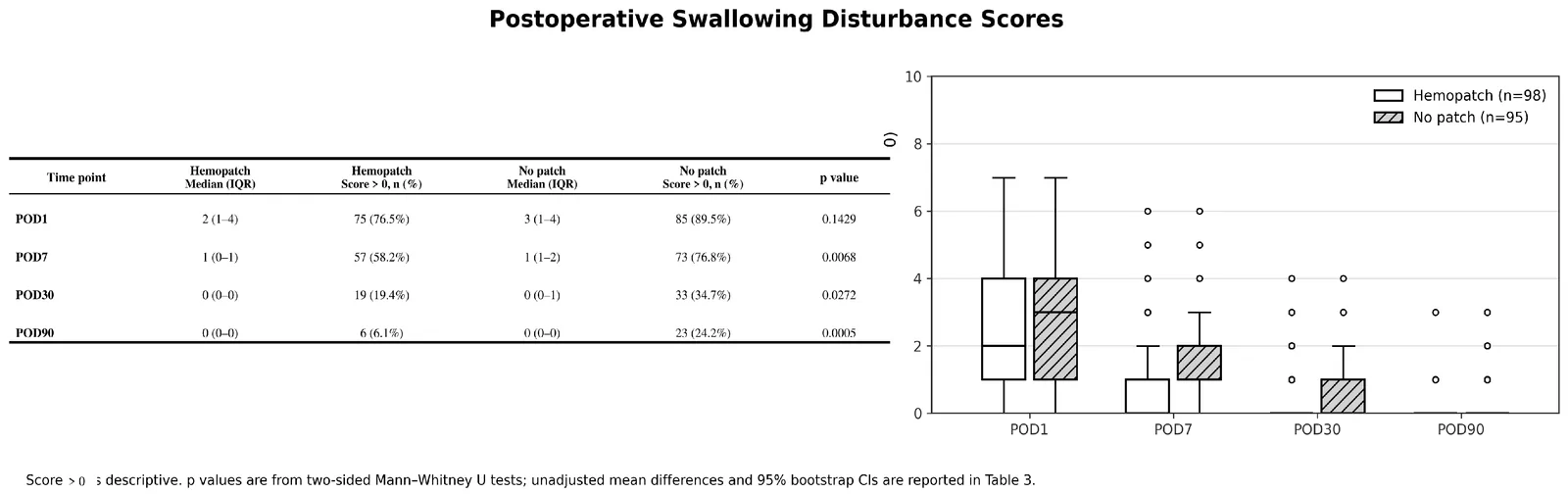
Postoperative swallowing disturbance scores during follow-up in the full unmatched cohort. The left summary reports median (IQR), the number (%) of patients with a score > 0, and the nominal between-group *p* value at each time point. Score > 0 is shown descriptively to make floor effects transparent; *p* values are from two-sided Mann–Whitney U tests using the full ordinal score distributions. Unadjusted mean differences with 95% bootstrap CIs are reported in Table 3. In the box plots, boxes represent the IQR, center lines represent medians, whiskers extend to the most extreme observations within 1.5 times the IQR, and points beyond the whiskers represent outliers. Hemopatch is shown as a white box and no patch as a hatched box. IQR, interquartile range.

Swallowing disturbance scores were also non-normally distributed and showed increasing floor effects during follow-up (Figure 2). On postoperative day 1, the unadjusted mean difference was −0.34 (95% CI −0.83 to 0.14; Mann–Whitney *p* = 0.1429). Patch use was associated with lower swallowing disturbance score distributions on day 7 (mean difference −0.35, 95% CI −0.67 to −0.03; *p* = 0.0068), day 30 (−0.17, 95% CI −0.38 to 0.05; *p* = 0.0272), and day 90 (−0.23, 95% CI −0.38 to −0.09; *p* = 0.0005). After Holm adjustment across the four swallowing time points, the adjusted *p* values were 0.1429, 0.0203, 0.0543, and 0.0018, respectively. The descriptive frequency of a score > 0 was 57 of 98 patients (58.2%) versus 73 of 95 (76.8%) on day 7, 19 of 98 (19.4%) versus 33 of 95 (34.7%) on day 30, and 6 of 98 (6.1%) versus 23 of 95 (24.2%) on day 90 in the patch and no-patch groups, respectively. These frequencies are provided to clarify the floor effect and were not used as separate inferential endpoints. Effect estimates are summarized in Table 3.

### 3.5. Scar Satisfaction

**Figure 3:**
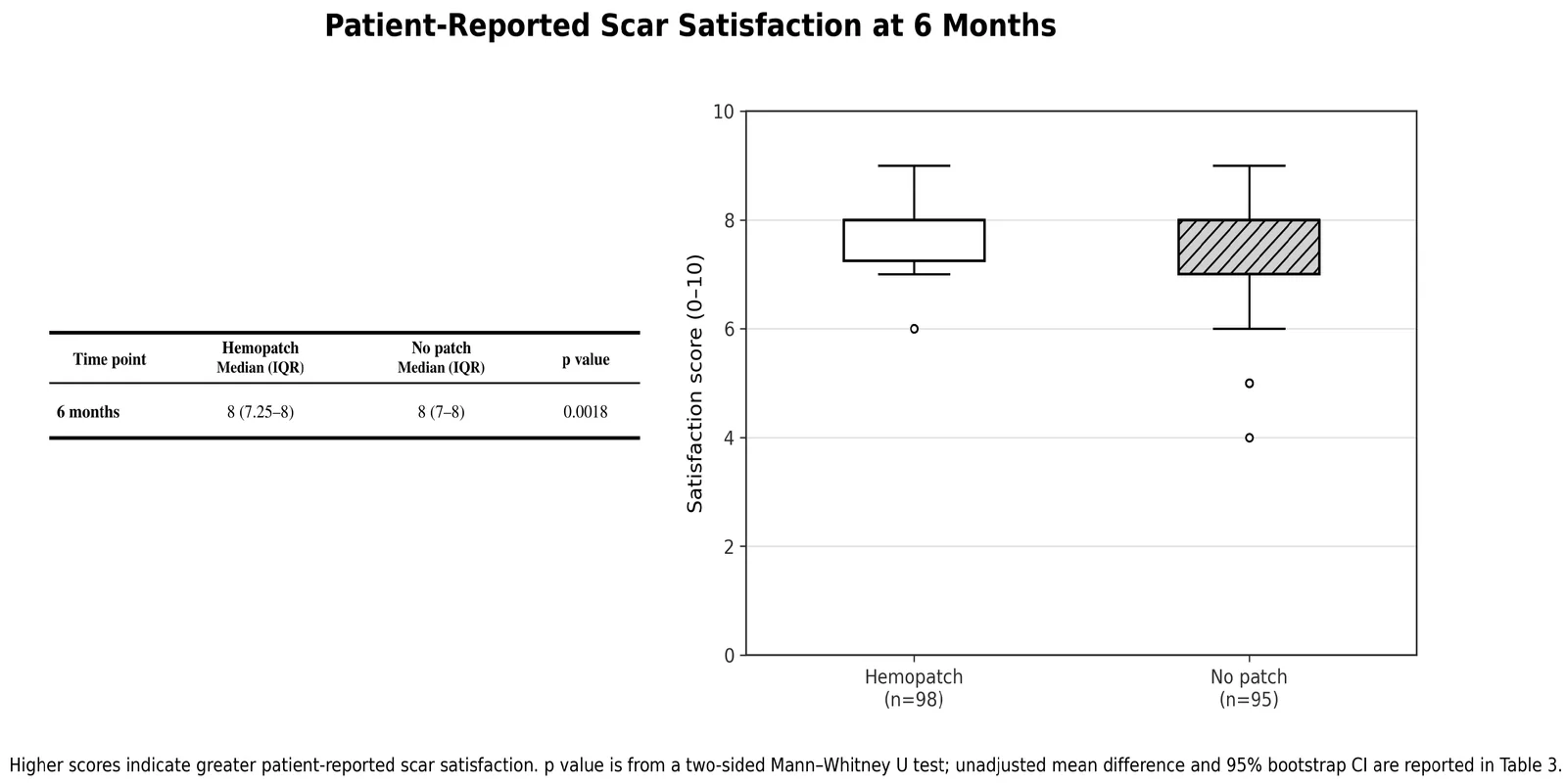
Patient-reported scar satisfaction at 6 months postoperatively in the full unmatched cohort. The left summary reports median (IQR) and the nominal between-group *p* value. The *p* value is from a two-sided Mann–Whitney U test using the full ordinal score distribution. The unadjusted mean difference with its 95% bootstrap CI is reported in Table 3. In the box plots, boxes represent the IQR, center lines represent medians, whiskers extend to the most extreme observations within 1.5 times the IQR, and points beyond the whiskers represent outliers. Hemopatch is shown as a white box and no patch as a hatched box. Higher scores indicate greater patient-reported satisfaction; this measure should not be interpreted as an objective scar-quality assessment. IQR, interquartile range.

At 6 months, patch use was associated with a shift toward higher patient-reported scar satisfaction scores in the full unmatched cohort (Figure 3). The unadjusted mean difference was +0.46 (95% CI +0.21 to +0.71; Mann–Whitney *p* = 0.0018). Median scores were 8 (IQR 7.25–8) in the patch group and 8 (7–8) in the no-patch group. Thus, the rank-based comparison indicated a distributional shift despite identical medians and substantially overlapping IQRs, emphasizing that the magnitude of the observed difference was modest. This outcome reflects patient-reported satisfaction on a 10-point VAS and should not Confirmed. Since “VAS” does not appear in the figure body or caption, the explanation “VAS, visual analog scale” can be removed. Please retain “IQR, interquartile range.”be interpreted as an objective assessment of scar quality or wound healing. The effect estimate is summarized in Table 3.

### 3.6. Adjusted Sensitivity Analysis

In the full unmatched cohort, separate proportional-odds ordinal logistic regression models adjusted for age, sex, operative time, intraoperative blood loss, and pathology showed associations between patch use and lower pain scores on POD7 (aOR 0.40, 95% CI 0.23–0.70; *p* = 0.0013; Holm-adjusted *p* = 0.0040) and POD30 (aOR 0.25, 95% CI 0.09–0.72; *p* = 0.0105; Holm-adjusted *p* = 0.0209). Lower swallowing disturbance scores were associated with patch use on POD7 (aOR 0.51, 95% CI 0.30–0.87; *p* = 0.0131; Holm-adjusted *p* = 0.0392), POD30 (aOR 0.48, 95% CI 0.25–0.93; *p* = 0.0289; Holm-adjusted *p* = 0.0578), and POD90 (aOR 0.19, 95% CI 0.07–0.51; *p* = 0.0010; Holm-adjusted *p* = 0.0040). Patch use was also associated with higher scar satisfaction at 6 months (aOR 2.37, 95% CI 1.35–4.16; *p* = 0.0026). No adjusted association was observed on POD1. The adjusted model for pain on POD90 could not be reliably estimated because all 98 patch-treated patients had a pain score of 0. These adjusted full-cohort results are sensitivity analyses and should not be interpreted as causal estimates or as a joint longitudinal model (Table 4).

## 4. Discussion

In this single-center retrospective cohort, the unmatched analysis of 193 patients found that collagen-based hemostatic patch use was associated with lower patient-reported pain and swallowing disturbance scores at selected postoperative time points and a distribution of scar satisfaction scores shifted toward higher values at 6 months. These associations were broadly supported by multivariable sensitivity analyses adjusting for measured baseline differences. However, the magnitude of the observed differences was small, and no statistically significant differences were observed in major postoperative complications.

The distributional characteristics of the patient-reported outcomes are important for interpreting these findings. At later follow-up, many patients in both groups reported no pain or swallowing disturbance, producing pronounced floor effects. Consequently, some statistically significant Mann–Whitney U tests occurred even when the medians and IQRs were identical, as seen for pain on days 30 and 90. The descriptive score > 0 frequencies in Figure 1 and Figure 2 show that residual symptoms were less frequent in the patch group, but these frequencies were not analyzed as separate binary endpoints. More broadly, both groups generally reported low symptom scores after the early postoperative period, and the study did not incorporate a prespecified minimally important clinical difference. The findings should therefore be interpreted as statistical associations in ordinal symptom distributions rather than evidence of a large or necessarily patient-perceptible clinical benefit.

The temporal pattern may nevertheless be informative. Differences were not evident on postoperative day 1 but appeared at later assessments. This timing is compatible with differences in local postoperative tissue response, minor oozing, or wound-bed stabilization [21,22], but the retrospective design cannot establish a biological mechanism. Mechanistic explanations should therefore be considered hypotheses for future prospective investigation rather than causal conclusions.

These findings should also be interpreted in the context of the recent literature questioning the overall clinical value of topical hemostatic agents in thyroid surgery. Previous meta-analyses have generally shown limited or inconsistent effects on conventional surgical endpoints such as reoperation for cervical hematoma, seroma formation, or other major postoperative complications [18,19], and a contemporary cohort study similarly found no clear reduction in thyroidectomy-related fluid complications [20]. Our results are consistent with the absence of a clear difference in uncommon complication-based outcomes and suggest that any potential association may be more readily detectable in patient-reported recovery measures.

Scar satisfaction requires particularly cautious interpretation. The 10-point patient-reported VAS used in this study was not a validated scar-specific instrument. Although the rank distribution differed statistically at 6 months, both groups had a median score of 8 and their IQRs overlapped substantially. The finding should therefore be described as a modest difference in patient-reported scar satisfaction rather than objective cosmetic improvement or evidence of superior wound healing. Future studies should incorporate validated instruments such as the Patient and Observer Scar Assessment Scale or Vancouver Scar Scale together with patient-reported measures.

Several methodological features strengthen the analysis, including a defined clinical cohort, standardized operative technique by a single experienced endocrine surgeon, repeated postoperative assessments, explicit assessment of distributional assumptions, rank-based analysis of markedly skewed VAS outcomes, and multivariable sensitivity analysis for measured confounding. At the same time, the single-surgeon design represents a tradeoff: it reduces variability in operative technique but limits generalizability to other surgeons, lower-volume settings, and institutions with different perioperative pathways.

This study has several important limitations. First, its retrospective and nonrandomized design is susceptible to selection bias, confounding by indication, and residual or unmeasured confounding; adjustment can address only measured and correctly specified covariates. The archived individual-level data underlying the originally submitted propensity-score-matched analysis could not be fully recovered. Therefore, the historical matched results could not be reproduced, and no propensity-score matching was performed in the revised analysis. The current analysis was restricted to 193 verifiable records from 296 potentially eligible patients, so selection bias related to data availability and cohort selection remains possible. Age and sex also remained imbalanced between groups.

Second, the sample size was limited to 98 Hemopatch and 95 no-patch patients, and major complications were uncommon. Thus, the study had limited power to assess rare safety outcomes or establish equivalence. Three Hemopatch records lacked hypoparathyroidism data, and no imputation was performed. Pain, swallowing disturbance, and scar satisfaction were assessed using subjective rating scales rather than validated quality-of-life or scar-specific instruments. Multiple symptom comparisons were performed; although Holm adjustment reduced several nominal associations, the findings remain exploratory. Finally, separate time-specific tests and ordinal regression models did not account for within-patient correlation across repeated assessments, and no joint longitudinal model was fitted. The single-center, single-surgeon design may also limit generalizability. Prospective multicenter studies using validated outcome instruments, prespecified clinically meaningful thresholds, explicit longitudinal models, and adequate power for safety outcomes are warranted.

## 5. Conclusions

In this retrospective cohort of 193 patients undergoing bilateral thyroidectomy, collagen-based hemostatic patch use was associated with modest differences in the ordinal distributions of several patient-reported recovery measures, including lower pain and swallowing disturbance scores at selected postoperative time points and a shift toward higher patient-reported scar satisfaction scores at 6 months. Pronounced floor effects were present at later follow-up, and the absolute magnitude and clinical importance of these differences remain uncertain. No statistically significant between-group differences in major postoperative complications were observed, but the study was not powered to establish comparative safety for uncommon events. These findings should be considered exploratory and require confirmation in prospective multicenter studies using validated outcome measures and prespecified thresholds for clinical importance.

## Figures and Tables

**Table 1:** Patient and operative characteristics in the full unmatched cohort.

Characteristic	Hemopatch (*n* = 98)	No Patch (*n* = 95)	*p* Value	SMD
Age, years	58.26 ± 12.13	55.19 ± 13.00	0.0921	0.244
Female sex, *n* (%)	79 (80.6)	70 (73.7)	0.3039	0.166
Operative time, min	61 (58–65)	61 (58–65)	0.8490	0.034
Intraoperative blood loss, mL	17.5 (15–20)	17 (15–20)	0.7397	0.050
Length of hospital stay, days	3 (3–3)	3 (3–3)	0.0470	0.285
Malignant pathology, *n* (%)	26 (26.5)	24 (25.3)	0.8707	0.029

Data are presented as mean ± SD, median (IQR), or *n* (%), as appropriate. Age was compared using Welch’s t-test; skewed continuous variables using the Mann–Whitney U test; and categorical variables using Fisher’s exact test. SMDs are absolute descriptive standardized mean differences for the full unmatched cohort; because no propensity-score matching was performed, they do not represent post-matching balance. Length of hospital stay is shown as a postoperative characteristic and was not included in the adjusted sensitivity models. SMD, standardized mean difference.

**Table 2:** Major postoperative complications in the full unmatched cohort.

Complication	Hemopatch *n*/*N* (%)	No Patch *n*/*N* (%)	Risk Difference (95% CI)	*p* Value
Hematoma	3/98 (3.1)	4/95 (4.2)	−0.0115 (−0.0638 to 0.0405)	0.7180
RLN injury	4/98 (4.1)	5/95 (5.3)	−0.0118 (−0.0740 to 0.0497)	0.7448
Hypoparathyroidism	14/95 (14.7)	15/95 (15.8)	−0.0105 (−0.1158 to 0.0947)	1.0000

Data are presented as *n*/*N* (%), where *N* is the number of evaluable patients. Risk differences are Hemopatch minus no patch; 95% CIs were obtained by percentile bootstrap resampling independently within each group (10,000 resamples). *p* values are two-sided Fisher’s exact tests. Three Hemopatch records lacked hypoparathyroidism data, yielding 95 evaluable patients in each group for that outcome. RLN, recurrent laryngeal nerve.

**Table 3:** Unadjusted effect estimates for patient-reported outcomes in the full unmatched cohort.

Outcome	Hemopatch Median (IQR)	No Patch Median (IQR)	Mean Difference (95% Bootstrap CI)	*p* Value	Holm-Adjusted *p*
Pain POD1	2 (1–3)	2 (2–3)	−0.19 (−0.66 to 0.27)	0.3814	0.3814
Pain POD7	0 (0–1)	1 (0–1)	−0.34 (−0.59 to −0.09)	0.0008	0.0023
Pain POD30	0 (0–0)	0 (0–0)	−0.11 (−0.22 to 0.01)	0.0223	0.0446
Pain POD90	0 (0–0)	0 (0–0)	−0.12 (−0.18 to −0.05)	0.0005	0.0022
Swallowing POD1	2 (1–4)	3 (1–4)	−0.34 (−0.83 to 0.14)	0.1429	0.1429
Swallowing POD7	1 (0–1)	1 (1–2)	−0.35 (−0.67 to −0.03)	0.0068	0.0203
Swallowing POD30	0 (0–0)	0 (0–1)	−0.17 (−0.38 to 0.05)	0.0272	0.0543
Swallowing POD90	0 (0–0)	0 (0–0)	−0.23 (−0.38 to −0.09)	0.0005	0.0018
Scar satisfaction at 6 months	8 (7.25–8)	8 (7–8)	+0.46 (+0.21 to +0.71)	0.0018	–

The primary patient-reported outcome analysis used the full unmatched cohort. Scores are reported as medians (IQR); mean differences are Hemopatch minus no patch and are provided as effect estimates with 95% percentile-bootstrap CIs based on 10,000 independent-group resamples. *p* values are two-sided Mann–Whitney U tests using the full ordinal distributions; because of tied scores, the asymptotic method was used. Holm-adjusted *p* values were calculated separately within the four pain and four swallowing comparisons. No patient-reported values were missing.

**Table 4:** Adjusted sensitivity analysis of patient-reported outcomes in the full unmatched cohort.

Outcome	Adjusted Common OR (95% CI)	*p* Value	Holm-Adjusted *p*
Pain POD1	0.81 (0.49–1.35)	0.4223	0.4223
Pain POD7	0.40 (0.23–0.70)	0.0013	0.0040
Pain POD30	0.25 (0.09–0.72)	0.0105	0.0209
Pain POD90	Not estimable *	–	–
Swallowing POD1	0.71 (0.43–1.18)	0.1842	0.1842
Swallowing POD7	0.51 (0.30–0.87)	0.0131	0.0392
Swallowing POD30	0.48 (0.25–0.93)	0.0289	0.0578
Swallowing POD90	0.19 (0.07–0.51)	0.0010	0.0040
Scar satisfaction at 6 months	2.37 (1.35–4.16)	0.0026	–

Adjusted common odds ratios were estimated from separate proportional-odds ordinal logistic regression models for each outcome and time point in the full unmatched cohort, controlling for age, sex, operative time, intraoperative blood loss, and pathology. For pain and swallowing, OR < 1 indicates lower odds of a higher symptom score; for scar satisfaction, OR > 1 indicates higher odds of a higher satisfaction score. Holm-adjusted *p* values were calculated separately within the pain and swallowing families. *p* values are reported to four decimal places. These models were not joint longitudinal models and did not account for within-patient correlation across time points. * Not estimable because all patients in the Hemopatch group had a postoperative day 90 pain score of 0, resulting in complete separation.

## Data Availability

The datasets generated and analyzed during the current study are not publicly available because of institutional data-protection and patient-privacy restrictions but are available from the corresponding author on reasonable request.
